# Why Sonochemistry in a Thin Layer? Constructive Interference

**DOI:** 10.1021/acs.jpcc.3c00804

**Published:** 2023-06-16

**Authors:** Daniel L. Parr IV, Chester G. Duda, Johna Leddy

**Affiliations:** Department of Chemistry, University of Iowa, Iowa City, Iowa 52240, United States

## Abstract

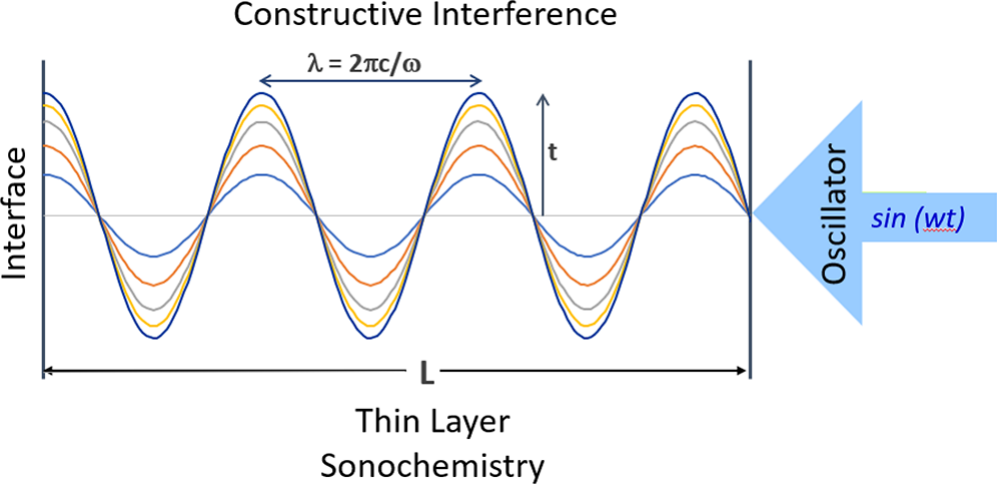

Sonochemistry in a thin fluid layer has advantages of no visible cavitation, no turbulence, negligible temperature changes (≲1 °C), low power transducers, and transmissibility (sound pressure amplification) of ≳10^6^. Unlike sonochemistry in semi-infinite fluids, resonance and so constructive interference of sound pressure can be established in thin layers. Constructive interference enables substantial amplification of sound pressure at solid fluid interfaces. Fluid properties of sound velocity and attenuation, oscillator input frequency, and thin fluid layer thickness couple to established resonance in underdamped conditions. In thin layer sonochemistry (TLS), thin layers are established where ultrasonic wavelength and oscillator–interface separation are comparable, about a centimeter in water. Solution of a one dimensional wave equation identifies explicit relationships between the system parameters required to establish resonance and constructive interference in a thin layer.

## Introduction

Sonochemistry is typically undertaken by irradiating a bulk fluid with ultrasound. Ultrasound is generated by a transducer at frequencies above human hearing, ≳ 20 kHz. In bulk fluids, ultrasound generates localized high temperatures (5000 K) and pressures (1000 atm, 1 atm = 101325 Pa).^1^ Where localized transient excursions of pressure and temperature are captured at interfaces, kinetic rates are increased. Ultrasound is exploited to impact chemical reactions and interfacial rates in syntheses of materials and nanomaterials, electrocatalysis, electrosynthesis, kinetics, analysis, deposition, and plating.^1−23^

**Figure 1:**
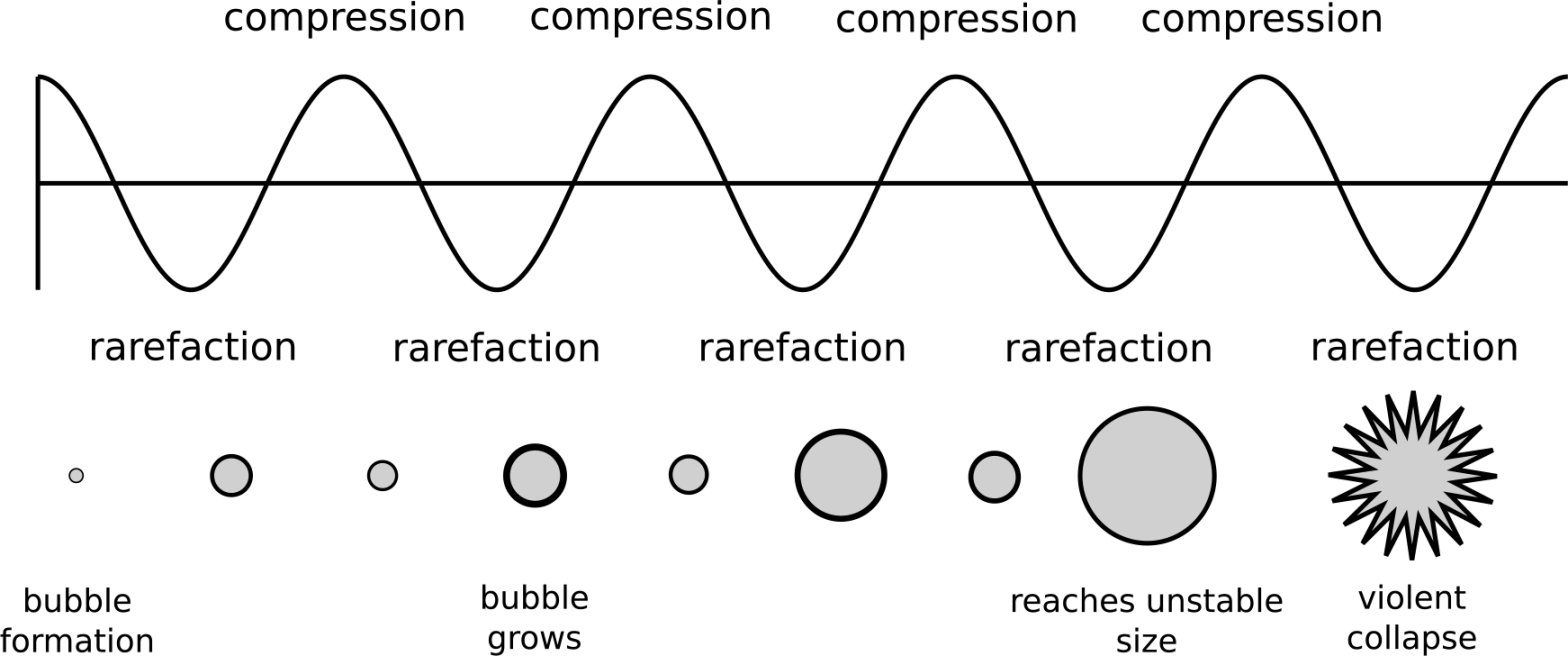
Sequential compression-rarefaction cycles are induced by irradiating bulk fluid with ultrasonic sound pressure oscillations. The sound pressure oscillations generate voids (bubbles) that grow with each cycle. At void collapse, extreme temperatures (>5000 K) and pressures (>1000 atm) transients are observed locally about the collapsed void.^1^ In bulk fluid, numerous void collapses in indiscriminate directions leads to chaotic cavitation. High power oscillators are needed to induce cavitation and heat in bulk fluids. The sketch is shown for a single input frequency.

In bulk fluids, sound pressure irradiation manifests in cavitation. Ultrasound is input from the transducer as a sinusoidal oscillation of sound pressure. The fluid undergoes compression and rarefaction under the pressure oscillations, see Figure 1. The compression–rarefaction cycle generates void volumes or bubbles within the fluid that grow with successive cycles. Once the voids reach a critical size on rarefaction, a following compression cycle collapses the void to generate high temperatures and pressures at the thin (≈0.1 μm) interface between the fluid and the void.^23^ Pressure and temperature transients in the bulk fluid arise from the localized energy release that generates cavitation. Cavitation is a chaotic process because as the voids collapse, energy is dispersed in indiscriminate directions. In classical sonochemistry (CS) undertaken in bulk fluids, substantial energy dissipates as heat in the fluid. CS requires high power transducers because transduction of input sound pressure to the fluid is inefficient.

**Figure 2:**
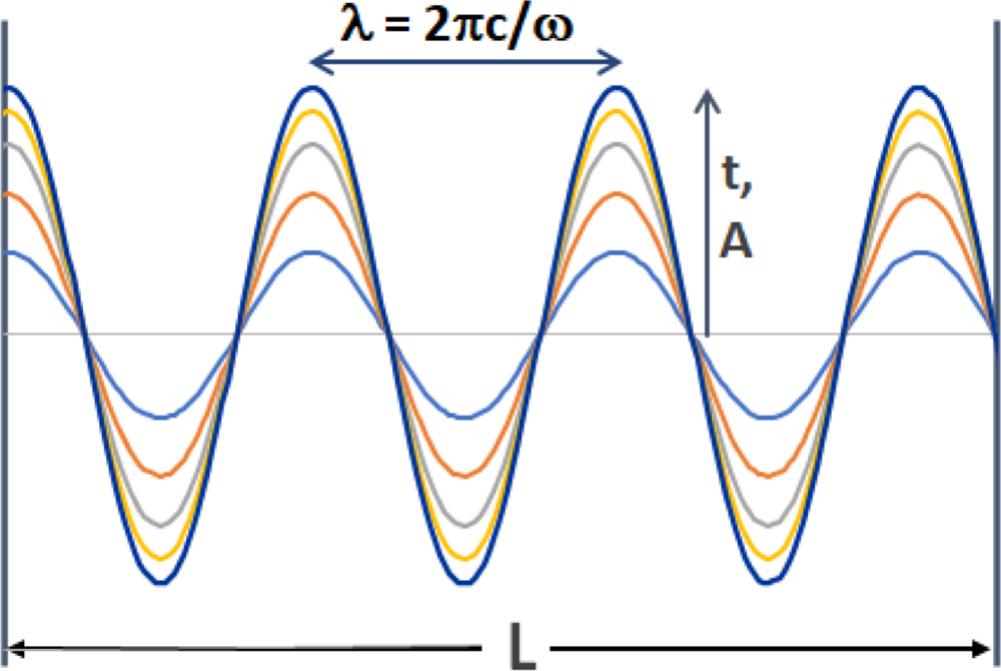
In a thin layer, constructive interference augments the sound pressure between boundaries separated by distance *L*. The amplitude of the sound pressure *A* grows with time *t* to reach a steady state. The sketch is shown for no flux of sound pressure through the interface (*u*_*x*_(0, *t*) = 0) with sinusoidal input at *x* = *L*. No attenuation due to properties of the fluid is illustrated. In real fluids, fluid properties damp sound pressure, so sound pressure is limited at a maximum amplification (transmissibility). The amplification of sound pressure *u*(*x*, *t*) is determined by radial frequency ω, distance between boundaries *L*, and speed of sound in the fluid *c*, as well as by sound pressure attenuation *k* and spatial attenuation coefficient α in the fluid. To establish resonance in a thin layer, the wavelength and thickness are roughly comparable.

Sonochemistry in a thin layer of fluid exploits constructive interference of the sound pressure oscillation,^24^ as sketched in Figure 2. In a well configured thin layer sonochemistry (TLS) experiment, the frequency (wavelength) of the sound pressure oscillations and the thickness of the thin layer are coupled to achieve resonance. On resonance, the amplitude of the oscillations builds as the sound pressure is additive on repetitive reflection between the transducer and the interfacial boundaries. The maximum amplification or transmissibility is limited by sound attenuation in the fluid.

Observed experimentally, where the reflective boundary is an electrode, several novel experimental outcomes are noted.^26,27^ The rates of slow interfacial (heterogeneous) electron transfer reactions are increased with no impact on mass transport. From voltammetry in an aqueous electrolyte, oxide layers on platinum electrodes are removed on extended sonication. Notably, during these experiments, where resonance is established,^24^ no cavitation is observed, perhaps because constructive interference focuses directional order across the fluid so that the localized, chaotic void collapses found in bulk fluids are avoided. The temperature of the fluid does not increase to within ≤1 °C, consistent with efficient energy transduction. A low power transducer such as a quartz crystal oscillator (QCO) is sufficient to establish constructive interference in a thin layer. QCOs consume little power and energy, as QCOs are driven by voltage with little current demand. QCOs are of high efficiency, commonly >90%. It is noted that construction of a TLS cell that effectively exploits construction interference is sensitive to the oscillator frequency ω, thickness *L*, and fluid properties required to achieve resonance.^24^ These experiments are undertaken voltammetrically, but the observations extend to interfacial chemical systems beyond electrochemistry, where constructive interference can be exploited in a thin layer to increase interfacial reaction rates. The paper provides a theoretical basis for establishing constructive interference in a thin layer. Model outcomes are consistent with the experimental observations.

Quantitative models for various aspects of sonochemistry in bulk fluids have been developed.^13,20−23,28−31^ Constructive interference has been noted for high power transducers in bulk fluids.^29−32^ However, in thin fluid layers where constructive interference can be exploited, sonochemistry is modeled very little. One paper reports models and experiments for ultrasound in thin layers with cavitation where resonance is not established.^30^ The sensitivity in construction of a thin layer sonoelectrochemical experiment that eliminates cavitation is noted.^24^

Here, a one-dimensional model of sound pressure *u*(*x*, *t*) is derived for sonochemistry in a thin layer. From the solutions to a wave equation, advantages of TLS over CS are identified. To establish constructive interference, the system must be underdamped to achieve resonance. Resonance is characterized by *u*(*x*, *t*), dependent on ω, *L*, and properties of the fluid and solid.^24,25^ The model is derived for conditions where no sound pressure is lost into the solid across the solid–fluid interface. There is a single input frequency ω. The model includes sound attenuation in the fluid, which sets maximum sound pressure delivered to the interface. Amplification at the interface is reported as transmissibility, the ratio of maximum sound pressure in the fluid to sound pressure input by the transducer. Transmissibility is found from *u*(*x*, *t*) in the thin layer. The mathematics establish why a thin layer is required to achieve resonance; identify the constraints for the coupling between ω, *L*, and fluid properties; provide the natural frequencies of the system ω_*n*_; and establish 2ω_*n*_ must be only slightly higher than the attenuation frequency *k*. Cumulative energy input to the interface is discussed. In heterogeneous chemical and electrochemical systems, TLS advantages over CS include lower power transducers (oscillators) for sound input and no obvious cavitation and temperature excursions. The model is consistent with TLS experiments demonstrated with an electrode as the reflective interface.^26,27^

## Description of the TLS System and Model Specification

**Figure 3:**
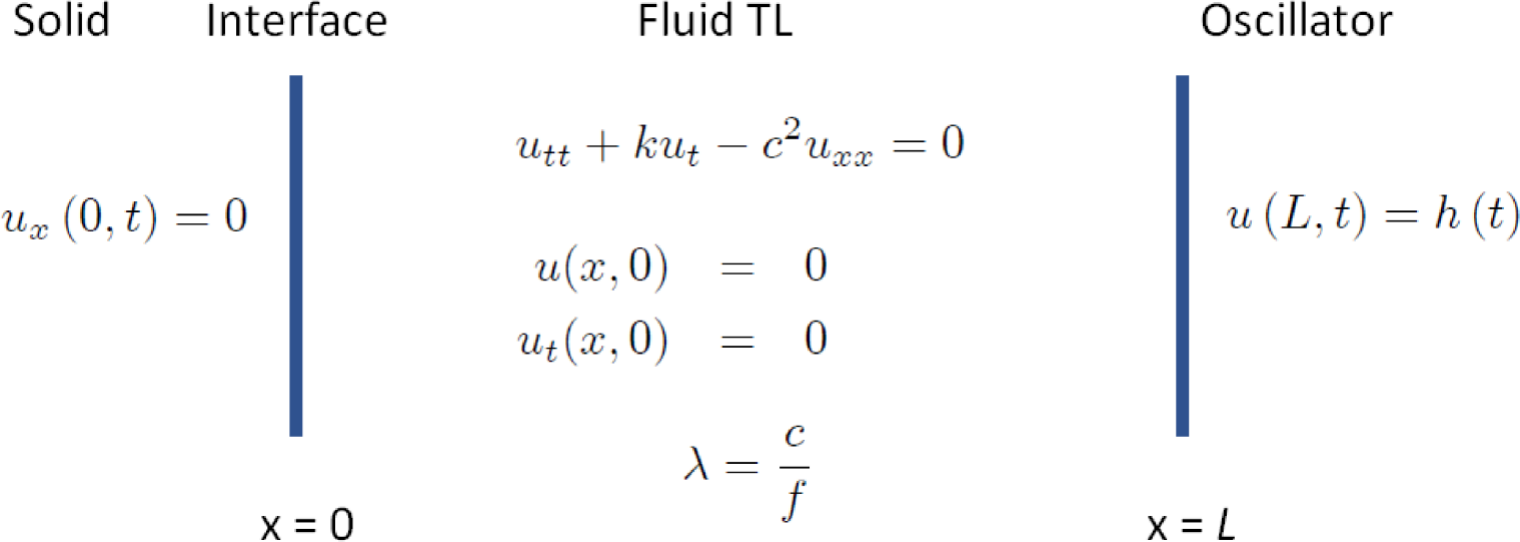
For sound pressure *u*(*x*, *t*), the conditions for the 1D, time- and space-dependent wave equation with temporal sound pressure attenuation *k* and acoustic velocity *c* in the fluid are shown. The oscillator inputs sound characterized by time (and ω) at *x* = *L*. Here, *h*(*t*) = sin(ω*t*). The sound pressure *u*(*x*, *t*) is amplified when constructive interference is established and the system is in resonance. Sound pressure captured at the solid–fluid interface *x* = 0 increases interfacial reaction rates. Amplification is reported as transmissibility, *T*, the ratio of maximum sound pressure in the fluid as compared to the input sound pressure at *x* = *L*. Parameters ω, *L*, *k*, and *c* set *T*.

The model system is sketched in Figure 3. Sound pressure input by the transducer at position *x* = *L* propagates as a wave toward the interfacial boundary at *x* = 0, where the sound pressure wave is reflected off the interface. The reflected wave is in phase with the incoming sound pressure wave to achieve resonance. Resonances allows constructive interference, so the amplitude of the wave builds additively over time. The increased amplitude manifests as increased transmissibility of the sound pressure in the thin fluid layer. Transmissibility measures the amplification of sound pressure in the fluid enabled by constructive interference. Fluid properties of acoustic velocity and attenuation rate limit the maximum amplitude. Reflectance at the interface is set by the acoustic impedance of the fluid and the interface.^24−26^ Acoustic impedance *Z* is the product of acoustic velocity and density in the phase, *Z* = ρ*c*.

### Parameters

Distance in one dimension is reported as *x* (m) and time is *t* (s). Oscillator frequency *f* (s^–1^) yields radial frequency 2*π f* = ω (rad s^–1^).

Energy and power are introduced by the transducer at *x* = *L*. Sound pressure *u*(*x*, *t*) (Pa) is input to the fluid at the face of the transducer (e.g., QCO) at *x* = *L* with time dependent transducer output pressure *h*(*t*) (Pa). Typically, the transducer outputs a sinusoidally oscillation at frequency *f*, or equivalently, angular frequency ω. In the ultrasonic range, *f* ≳ 20 kHz. The transducer frequency and the acoustic velocity of the fluid *c* (m s^–1^) set the wavelength λ (m) of the sound wave in the fluid.
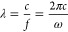
1For water, *c* = 1482 m s^–1^ at 20 °C. Wavelength λ of 1.00 cm is established for *f* of 148.2 kHz. For the typical sonochemical range of 20 to 100 kHz, λ falls between 7.4 and 1.48 cm, and λ is on the order of centimeters. Medical imaging frequencies are in the GHz range with wavelengths on the order of micrometers.

Transmissibility *T* measures the amplification in the fluid as maximum sound pressure generated in the fluid relative to the sound pressure input at *x* = *L*. Where sound is amplified in the fluid, *T* > 1 is characteristic of constructive interference.

Transmissibility is limited by the rate sound pressure attenuates in the fluid, *k* (rad s^–1^). For a natural frequency of the system ω_*n*_, amplification is only possible for underdamped systems, where *k* < 2ω_*n*_. The model finds ω_*n*_ and the relationship between ω_*n*_ and *L*. The temporal attenuation rate *k* is proportional to the spatial attenuation coefficient α (m^–1^).
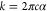
2In liquids and solids,^2,33−35^ α is well approximated from the fluid properties of dynamic viscosity η (Pa s) and density ρ (kg m^–3^). Kinematic viscosity (m^2^ s^–1^) is ηρ^–1^.
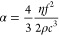
3For water at 20 °C, η is 8.90 × 10^–4^ Pa s and ρ is 1000 kg m^–3^ such that α = 1.823 × 10^–16^ (m^–1^ s^2^) *f*^2^. At 22 kHz, α is 87.2 nm^–1^ and *k* is 8.18 × 10^–4^ rad s^–1^. Amplification marks resonance and so constructive interference. Amplification is measured as transmissibility *T* that is found from *u*(*x*, *t*) evaluated in the model.

### Specification of the Modified Wave Equation

Propagation of sound pressure *u*(*x*, *t*) characterizes the response of the thin layer sonochemical system through the one dimensional wave equation (*u*_*tt*_ = *c*^2^*u*_*xx*_) modified for sound attenuation at rate *u*_*t*_. *u*_*t*_ is the first derivative in time. *u*_*tt*_ and *u*_*xx*_ are second derivatives.
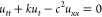
4The system is bounded for 0 < *x* < *L* and *t* ≥ 0. The system is specified with two boundary and two temporal conditions. The boundary at *x* = 0 is completely reflective, and there is no flux of sound pressure into the phase at *x* < 0.
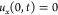
5The transducer drives the sound pressure with input sound pressure *h*(*t*) at *x* = *L*.
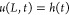
6At *t* = 0, initial conditions are that sound pressure in the fluid is zero and the time rate of change of sound pressure is zero.
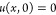
7
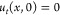
8

#### Model Assumptions and Limitations

Assumptions of the model are several. The model is linear across one spatial dimension, *x*. The surfaces of the transducer and the reflective interfaces have large, flat cross sections and the surfaces are parallel. The attenuation coefficient *k* is a constant, *x* and time *t* independent. Fluid properties are homogeneous and invariant across 0 < *x* < *L*. No heat is dissipated in the fluid. The interface at *x* = 0 is totally reflective (eq 5) and no sound pressure permeates into the interface. At *x* = *L*, the transducer inputs a single sinusoidal wave. Here, at a fixed frequency ω and unit amplitude, *h*(*t*) = sin(ω*t*) (eq 10).

The most substantial limitation of the model is that the interface is 100% reflective. In practice, ultrasound is lost to the solid. Reflectivity of the interface varies with combinations of solid and fluid, as detailed in ref (24). For example, in water, tungsten, silver, and graphite reflect 97.1%, 89.7%, and 38.5%, respectively. The reflective boundary condition of eq 5 is an appropriate first approximation, but will be less effective where reflectivity is lower. To account for loss of sound pressure into the reflective solid, the no flux boundary condition of eq 5 would be replaced with *u*_*x*_(0, *t*) = *a*, where *a* is the fraction transmitted (lost) into the solid. Where sound pressure is lost into the solid, the magnitude of the constructive interference is diminished. In practical systems, the sound pressure transmitted into the solid may dissipate as heat. Experimentally, Duda observed small losses of silver from silver electrodes and disintegration of graphite pencil electrodes on sonication at 20 kHz.^26,27^ Fluid temperatures measured with more reflective electrodes are unchanged.

In practice, the transducer may output multiple frequencies at varying amplitudes, and the faces of the transducer and reflective surface may not be strictly parallel. The single sinusoidal input *h*(*t*) suffices to characterize the oscillator, although unanticipated complexities of multiple frequencies may arise. Dependent on parameters that include *L*, λ, ω, *k*, and reflectivity, construction of the thin layer cell is somewhat delicate.^24^

Despite the limitations of the model assumptions, the outcomes of the model in underdamped conditions are consistent with the experimental results for sonoelectrochemistry undertaken in a thin layer configuration absent cavitation.^26,27^

### Solution

The problem is re-expressed as
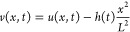
9

The input from the oscillator *h*(*t*) is specified as a simple sine wave.
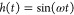
10

The one-dimensional partial differential equation is solved as detailed in the Appendix, with additional details in the Supporting Information.^25^

The solution for the sound pressure is found in *v*(*x*, *t*), as developed in eq A.17. *v*(*x*, *t*) is expressed with various parameters as the sum of two infinite series with index *n*.
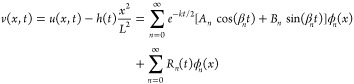
11The first term on the right largely characterizes the impact of input at the surface of the oscillator, where the exponential term captures damping by attenuation in the fluid as *t* → ∞. The second term characterizes compounding of the sound pressure oscillations over time coupled with damping due to attenuation. All parameters subscripted with *n* evolve with the series index *n*, where *n* is an integer, *n* ≥ 0.

ϕ_*n*_(*x*) is the time-independent, cosine function that describes the standing waves established in the fluid. This arises from the assumption that *v*(*x*, *t*) is represented as a series that is the product of separable time *v*_*n*_(*t*) and space-dependent ϕ_*n*_(*x*) terms, eq A.8. Defined in eq SI.2, .

β_*n*_ is a frequency that relates the natural frequency of the thin layer ω_*n*_ to the temporal attenuation coefficient *k*. From eq A.13, . Amplification requires underdamped systems where *k* < 2ω_*n*_, so amplification requires β_*n*_ > 0. Constructive interference is best established as β_*n*_ → 0. At β_*n*_ = 0, the system is critically damped and constructive interference is not established. For a given fluid, β_*n*_ is fixed.

*A*_*n*_ characterizes the amplitude of the cosine term of eq 11, dependent on the initial oscillation input at the oscillator face *h*(0) and *r*_*n*_, where *r*_*n*_ is defined in eq SI.13.
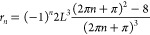
12From eq SI.15*A*_*n*_ = −2*h*(0)*r*_*n*_*L*^–3^. *A*_*n*_ is independent of *L*.

*B*_*n*_ characterizes the amplitude of the sine term, dependent on *r*_*n*_, the initial oscillation at the oscillator face *h*(0) and the rate of change (the frequency) of the oscillation at the oscillator face *h*′(0). Equation 10 defines *h*(*t*). Equation SI.17 defines β_*n*_*B*_*n*_ = −*r*_*n*_*L*^–3^[2*h*′(0) + *kh*(0)]. *B*_*n*_ is also independent of *L*. As β_*n*_ decreases, *B*_*n*_ increases.

*R*_*n*_(*t*) characterizes the compounding of the sound energy by constructive interference, with acknowledged limits due to attenuation. This is detailed in the SI, sections SI.1.4 and SI.1.5. Because the integrals for *R*_*n*_(*t*) do not yield to immediate solution, *R*_*n*_(*t*) is evaluated numerically.

The model was evaluated numerically using Python and Julia. COMSOL is an alternative and would be needed for more complex geometries and conditions. The approach through solution of PDEs does provide several explicit numerical relationships between system parameters that maximize *T*.

**Figure 4:**
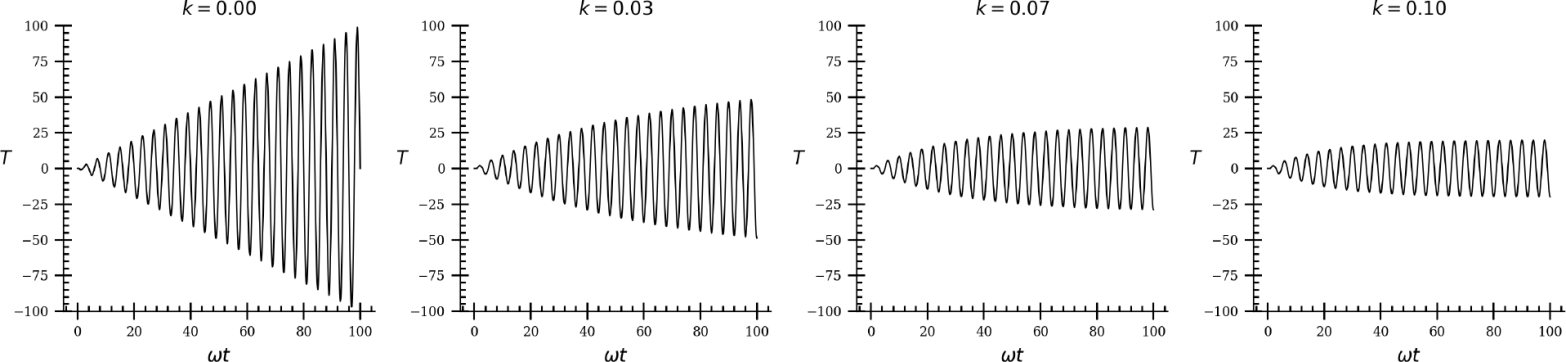
Transmissibility *T* with dimensionless time ω*t* is shown as attenuation *k* (rad s^–1^) increases. When *k* = 0, there is no attenuation of sound pressure in the fluid and *T* grows without bound as ω*t* increases. For *k* > 0, *T* comes to steady state as ω*t* increases. The maximum *T* decreases as *k* increases because increased attenuation suppresses sound pressure amplification. For *k* = 0.10, *T* approaches 20. For lower *k*, *T* is greater. In water near 25 °C, *k* = 0.000818 rad s^–1^ and limiting amplification *T* is 3.65 × 10^6^. Steady state amplification is achieved rapidly, by ω*t* ≲ 100. For ω of 22 kHz, steady state is reached <5 ms.

Transmissibility is evaluated from the maximum sound pressure and the sound pressure input at *x* = *L*. Transmissibility is shown as a function of ω*t* in Figure 4 for no attenuation and for temporal attenuation constants 0.03 ≤ *k* ≤ 0.10 rad s^–1^. In common solvents, *k* ≲ 0.01 rad s^–1^ for *T* of the order of 10^6^.^24^

Each oscillation builds energy at the interface. Kinetic rates of interfacial reactions are increased by energy deposited at the interface by high frequency sound pressure oscillations.

## Discussion of Model Outcomes

The model yields *u*(*x*, *t*). When constructive interference is established, the system is in resonance and *T* > 1. Transmissibility is amplification, the ratio of the maximum sound pressure achieved in the fluid to sound pressure input by the oscillator. Several observations about TLS are drawn from the solution for *u*(*x*, *t*) in eq 11. Also outlined are what parameters maximize *u*(*x*, *t*) and *T* and how TLS without cavitation compares to CS in practical terms.

Several inferences about sonochemistry in thin layers and under semi-infinite conditions are drawn for the solution for sound pressure. The solution is discussed. Distinctions between TLS and CS are presented.

### Thin Layers: No Cavitation

In part, the form of ϕ(*x*) identifies distinctions in CS and TLS. The general form of the solution for  in eq SI.4 is equivalently expressed as  = . For thin layer conditions, *x* is restricted to a range 0 to *L*, so ϕ_*n*_(*x*) is bounded and sound pressure waves are limited in amplitude. Under appropriate conditions for λ, the system comes into resonance and sound pressure is amplified (Figure 4) by constructive interference (Figure 2). In the semi-infinite conditions of CS, however, *x* is not bounded and the magnitude of *x* can grow large. With a semi-infinite boundary condition, no reflective thin layer boundary is specified at the interface ϕ_*n*_^′^(*x* = 0) = 0, and resonance can not be established.

**Figure 5:**
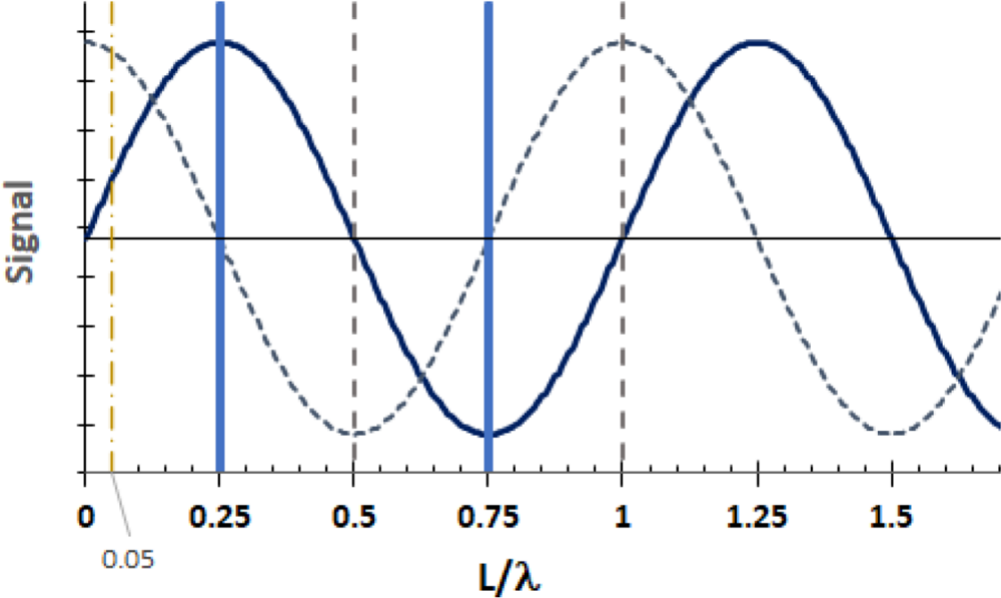
For sinusoidal ultrasound *u*(*x*) input at *L*/λ = 0, sound pressure (black line) propagates into the fluid. For the thin layer formed with the reflective wall well placed at *L*/λ = 1/4 (vertical blue line), the sound pressure *u*(*x*) is at a maximum at *L*/λ = 1/4, and the constraint that sound pressure is reflected from the surface *u*_*x*_ = 0 is met. The sound pressure flux *u*_*x*_(*x*) at each *x* is shown by short blue dashes. On reflection at *L*/λ = 1/4, reflected sound pressures add to incoming sound pressure to achieve constructive interference. Conditions for solution to model are met for *L*/λ = (2*n* + 1)/4, shown by vertical blue lines at *L*/λ of 0.25 and 0.75. For the wall poorly placed at *L*/λ = 0.5, *u*(*x*) = 0 and *u*_*x*_(*x*) < 0. Sound pressure at the wall is negligible, and *u*_*x*_ < 0 means any sound reflected at the wall reflects with a sign opposite the incoming wave, so the incoming and reflected waves cancel and destructive interference arises. A similar cancellation of incoming and reflected waves and destructive interference occurs at *L*/λ = 1.

The model identifies constructive interference is established for underdamped conditions.
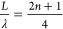
13Conditions for constructive interference are illustrated for *n* = 6 in Figure 2. In Figure 5, for a given instant in time and frequency, sound pressure input at *L*/λ = 0 propagates into the fluid. If the solid reflective interface is at *L*/λ = (2*n* + 1)/4, sound pressure is reflected at the interface straight back toward the oscillator and the incoming and reflected waves add to generate constructive interference across the thin layer. The waves add as the condition *u*_*x*_(*L*/λ = (2*n* + 1)/4) = 0 and the reflected sound pressure wave overlays the incoming wave. Where the interface is ill-positioned at *L*/λ = (2*n*)/4, sound pressure is negligible and *u*_*x*_ < 0. The little sound pressure reflected at the interface near *L*/λ of *n*/2 is of the opposite sign of the incoming wave, and the incoming and reflected waves cancel to yield destructive interference. For the semi-infinite systems common in CS, where *k* is underdamped, there is no reflective boundary to generate constructive interference and no amplification. In real systems, the sound pressure would dissipate at distances sufficiently far from the oscillator because underdamped conditions *k* < 2ω_*n*_ are only achieved as *n* → ∞, as *L* → ∞.

### Thin Layers: With Cavitation

Within the one-dimensional model developed here, fluid properties are uniform and input sound pressure is sinusoidal of low intensity. No cavitation is found either for this model in underdamped conditions or for TLS experiments where resonance and constructive interference are established.^26,27^ Moussatov, Granger, and Dubus^30^ developed a two-dimensional model for a thin fluid layer at a cylindrical horn transducer with cavitation, where eq 13 is not satisfied. From their models and experiments, substantial sound pressure amplification is observed. Cavitation is generated for cell thickness (*L*) “much smaller” than the acoustic wavelength (2π*c*/ω). Both linear components along the principle axis and radial components about the horn tip contribute to amplification.

It is not clear whether constructive interference is critical to amplification in a thin layer with cavitation. Considered in the simple one-dimensional framework in Figure 5, yellow dashes at *L*/λ = 0.05 mark *L* much smaller than λ, where *u* > 0 and 0 < u_*x*_ < 1. If a reflective boundary were established at *L*/λ = 0.05, some fraction of the reflected wave would add to the incoming wave, but *u*_*x*_ ≠ 0 conveys that sound pressure also travels beyond *L*/λ = 0.05. In real systems, these conditions are not sufficient to establish cavitation or identify constructive interference as a major component of amplification in thin layers with cavitation. However, amplification is clearly demonstrated in experiments and models for thin layer systems with^30^ and without^26,27^ cavitation.

### Criticality of Near Zero β_*n*_ for *k* Underdamped

The counterbalance of the attenuation damping exp[−*kt*/2] by β_*n*_ is captured in eq 11. The parameters ϕ_*n*_(*x*) and *A*_*n*_ are independent of *k* and β_*n*_. But, *B*_*n*_ increases as β_*n*_ decreases, where *B*_*n*_ ∝ β_*n*_^–1^. The coefficient of the sine term under the first summation on the right-hand side is proportional to β_*n*_^–1^ exp[−*kt*/2]. From section SI.1.4, *R*_*n*_(*t*) convolutes exp[−*kt*/2] and β_*n*_, but the general pattern remains as a ratio β_*n*_^–1^ exp[−*kt*/2]. The ratio β_*n*_^–1^ exp[−*kt*/2] characterizes both summations. If time-independent β_*n*_ does not approach zero, then exp[−*kt*/2] will tend to damp *u*(*x*, *t*) as *t* increases. Thus, to achieve resonance and amplification, β_*n*_ → 0 is needed. One condition to achieve resonance is an underdamped system where 2ω_*n*_ > *k*, consistent with β_*n*_ → 0.

Solutions here are for underdamped systems specified in eq A.13, , where ω_*n*_^2^ is larger than *k*^2^/4. For effective amplification through resonance, ω_*n*_ just slightly higher frequency than *k*/2 is required for β_*n*_ → 0. That is, twice the natural frequency ω_*n*_ should slightly exceed the temporal attenuation coefficient. The optimal frequency of oscillations under resonance should just suffice to overcome the attenuation frequency.

### Criticality at β_*n*_ = 0 for *k* Critically Damped

Solutions for underdamped conditions establish that β_*n*_ approaching zero from the positive side. This establishes constructive interference without cavitation. Critically damped conditions occur when β_*n*_ = 0.

Eq 11 describes sound pressure in two series that both embed standing waves ϕ_*n*_(*x*). The first series includes input from the oscillator coupled with attenuation damped exponentially in time. The second series includes compounding of pressure oscillation with attenuation. ϕ_*n*_(*x*) is independent of β_*n*_. In the first series, for β_*n*_ = 0, sin(β_*n*_*t*) = 0, and cos(β_*n*_*t*) = 1. *A*_*n*_ is finite, independent of β_*n*_. The first series is suppressed by exponential term exp[−*kt*]. For the second series for β_*n*_ = 0, *R*_*n*_(*t*) scales as β_*n*_^–1^ (eq A.18). *R*_*n*_(*t*) increases without bound, where β_*n*_ = 0 and *v*(*x*, *t*) tends to infinity. The critically damped conditions where β_*n*_ = 0 do not lead to the constructive interference absent cavitation in the thin layer. Whether critically damped conditions lead to cavitation is not determined from the model.

### Impact of *h*(*t*) across *L* ≤ *x* ≤ 0

From eq 11, the sound pressure *u*(*x*, *t*) in a thin layer is constrained by the two summations. *u*(*x*, *t*) varies across the thin layer with *h*(*t*), the sinusoidal input from the oscillator at *x* = *L*. But, the impact of *h*(*t*) on *u*(*x*, *t*) diminishes parabolically as *x* approaches zero. At the interface (*x* = 0), *v*(0, *t*) = *u*(0, *t*). On input at *x* = *L*, *h*(*t*) induces sound wave oscillations in the fluid. The induced oscillations establish sound pressure at the interface (*x* = 0). Where resonance is achieved, *T* > 1 and sound pressure is increased at *x* = 0.

### The Solution

The principle outcome for solution of eq 4 for the thin layer boundary and initial conditions of eqs 5–8 is the roots of the equations.
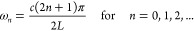
14Equation 14 relates the natural frequency of the cell ω_*n*_ to the distance between the oscillator and the interface *L* and the sound velocity in the fluid. Multiple roots are provided by *n* where all values of *n* provide valid roots. In practice, large *n* may not be practicable. Resonance is achieved where eq 14 is met. In semi-infinite systems as *L* → ∞, ω_*n*_ approaches zero for finite *n*.

The wavelength of ultrasound, λ = 2π*c*ω^–1^. To achieve constructive interference in a thin layer, conditions require λ = 4*L*(2*n* + 1)^−1^ (eq 13). The wavelength can be no greater than 4*L* and only the specific values λ*L*^–1^ = 4(2*n* + 1) ^–1^ will provide resonance. Given practical limitation on *n*, λ*L*^–1^ falls between 4 and about 4/7 to 4/9.

To satisfy β_*n*_ approaching zero, greatest resonance is reached where at least one experimentally accessible ω_*n*_ is slightly greater than *k*/2. From eqs 2 and 3, ω_*n*_ slightly greater than 3πρ*c*^2^η^–1^ is needed.

The impact of *k* on *T* is shown in Figure 4. For no attenuation in the fluid, *k* = 0 and the *T* grows without bounds. As *k* increases, *T* comes to steady state, where maximum *T* decreases as *k* increases. The amplification of sound pressure is substantial at about 50-fold for *k* = 0.03 and about 20-fold for *k* = 0.10. Values of *k* for common solvents are ≲0.01 rad s^–1^, with *T* ≳ 2 × 10^5^. Where *k* > 0, the system approaches steady state by ω*t* of 100. Steady state is achieved rapidly as ω is high. For ω ≳20 kHz, time to steady state is less than 5 ms. At steady state, each oscillation builds energy at the interface. At high frequencies, the accumulated energy can impact interfacial rates.

The amplification is dependent on the fluid properties. For water at 25 °C, *k*_water_ = 8.18 × 10^–4^ rad s^–1^ and *T*_water_ = 3.65 × 10^6^. From the model, maximum transmissibility and temporal sound pressure attenuation are inversely proportional, *Tk* = *k*_water_*T*_water_ = 2986 rad s^–1^. For a given solvent, *Tk* is constant,^24^ and transmissibility is maximized where solvent-specific *k* is minimized. In common solvents, *T* is on the order of 10^6^ with constructive interference and no cavitation.

Note that thickness is defined as *L* = (2*n* + 1)λ/4 (eq 13). For a given λ, multiple *L* values are specified. At these values, *L* optimally reflects sound pressure into the fluid to achieve constructive interference (Figure 5). For *n* of 0 or 1, *L* is 0.25 λ or 0.75 λ, and *L* is less than λ. In CS, typically *L* > λ. As *n* increases, the thickness increases in discrete increments. For λ on the order of centimeters, single digit values of *n* are practicable. From eq 1, oscillator frequency tunes λ. If *L* is fixed, tuning λ may allow constructive interference to be established in confined gaps.^24^ Frequencies of gigahertz map to thicknesses on the order of micrometers.

### Comparison of TLS and CS in Practice

Sonochemistry in a thin layer establishes constructive interference when the thin layer system is in resonance. Without resonance, there is no amplification. In a thin layer of thickness *L*, an oscillator at *x* = *L* inputs a sinusoidal signal *h*(*t*) that transmits across the layer to induce oscillations. Resonance is established when sound pressure *u*(*x*, *t*) reflected at the interface (*x* = 0) adds constructively across the cell. Resonance and amplification require parameters of ω_*n*_, *L*, *k*, and *c* meet conditions specified by the model in eqs 14 and A.13. The amplification is reported as transmissibility, the ratio of the maximum sound pressure in the fluid to the input sound pressure set by *h*(*t*). The objective is to maximize amplification, *T* > 1.

Practical constraints of how to construct a successful thin layer cell are presented in ref (24). This includes information about the materials parameters for common fluids and common solids that form the interface. Reflectance at the fluid solid interface is set by the acoustic impedance of the fluid and solid phases. Reflectance is (*Z*_2_ – *Z*_1_)(*Z*_2_ + *Z*_1_)^−1^. For example, reflectance at the water platinum interface is high 95% but lower for the water graphite interface, 38.5%. It is noted that construction of TLS systems is more delicate than construction of CS systems.^24^

### Distinctions in TLS and CS

Resonance can be established in a thin fluid layer but not in a semi-infinite homogeneous fluid. Amplification by constructive interference can be achieved in TLS. For TLS with the oscillator at *x* = *L* and the interface at *x* = 0, the condition *u*_*x*_(0, *t*) = 0 sets the reflection of sound pressure at the interface that is needed to establish resonance. In CS, where effectively *L* is infinitely large, the boundary condition *u*_*x*_(0, *t*) cannot be set. Attenuation would damp oscillations across the fluid and conditions of 2ω_*n*_ slightly greater than *k* and ω_*n*_ = *c*(2*n* + 1)π(2*L*)^−1^ can not be simultaneously satisfied as *L* → ∞. Commonly in CS, resonance is not established in the bulk fluid and *k* damps input sound pressure over distance.

In underdamped systems, constructive interference is established where β_*n*_ → 0. In critically damped systems, β_*n*_ = 0 and constructive interference is lost. Whether critical damping leads to cavitation can not be determined from the current model.

Resonance established in TLS requires a lower energy oscillator (e.g., QCO) than used in CS (e.g., sonic horn). The model is developed for input *h*(*t*) = sin(ω*t*), but higher amplitude generated by higher intensity oscillators are anticipated to further enhance amplification in the system provided the constraints on ω_*n*_, *L*, *k*, and *c* are met. Increased impacts of oscillator intensity at electrodes in TL electrochemistry experiments have been observed.^26,27^ Because the input energy is lower in TLS, the hardware is simpler and electrical energy consumption and cost are lower. The waste heat in a TLS is negligible as cell temperature changes ≤1 °C over extended time of sonication.^26,27^ No cavitation is observed.

Resonance is maximized at steady state. Because ω is high, TLS systems rise to steady state rapidly. This is observed experimentally.^26^

The rate pressure is delivered to the interface (*x* = 0) is power. For resonance at ω_*n*_, energy is delivered with each oscillation. Integration of the power over time yields the energy delivered to the interface. For an oscillator such as a QCO, the energy delivered is about 1 kJ mol^–1^ s^–1^. For a 20 kHz transducer, ≈0.05 J mol^–1^ reaches the interface with each oscillation. Sonication over longer time increases energy at the interface. Evidence of accumulated energy is found for removal of oxide from platinum in water over about 6–10 min.^26^

Within the context of the model, an important caveat is that the interface is perfectly reflective and no energy is lost to the solid at the interface. The model accounts fluid properties but assumes a reflective interface with no flux of sound pressure across interface into solid at *x* = 0. From a bulk perspective, sound travels more efficiently in denser media. Reflectivity of the interface is important. Solid fluid combinations with high reflectivity are reported in reference.^24^ In TLS experiments, carbon electrodes such as pencil leads have disintegrated and silver wires used as references lost enough silver to deposit silver on the platinum electrodes.^26^ To account for loss of sound pressure into the solid, the no flux boundary condition *u*_*x*_(0, *t*) = 0 is replaced, which would perhaps require strictly numeric solution as with COMSOL.

By appropriate configuration to establish resonance, it may be possible to control the energy at the interface to overcome the activation energy of interfacial reactions. Energy input over time accumulates with oscillations, provided the appropriate conditions are met to establish constructive interference in a thin fluid layer.

## Synopsis

Why sonochemistry in a thin layer? Constructive interference amplifies sound pressure.

The model of the wave eq (eq 4) embeds properties of the fluid *k* and *c*, properties of the oscillator ω, and separation distance *L* between the oscillator at *x* = *L* and the perfectly reflective solid fluid interface at *x* = 0. Constructive interference is not established unless the system is in resonance. At resonance, natural frequencies ω_*n*_ are established by *L*, *c*, and ω (eq 14). Attenuation damps propagation of sound pressure in the fluid. To overcome the damping, 2ω_*n*_ must be very slightly greater than *k* as β_*n*_ → 0 for underdamped conditions (eq A.13) Amplification of sound pressure by constructive interference is reported as transmissibility *T*. For common solvents, *T* is of the order of 10^6^. Energy is delivered to the interface at ≈1 kJ (mol s)^−1^. From the model equations, constructive interference can not be established in bulk fluids of semi-infinite extent. Constructive interference is established in thin fluid layers for underdamped conditions where ultrasonic wavelength λ and *L* are comparable, 4 ≤ λ/*L* ≲ 0.5. In critically damped systems, (β_*n*_ = 0), constructive interference is not established.

The model^25^ is vetted against experiments in TLS, where resonance and constructive interference are established,^26,27^ and there is no visible cavitation and no turbulence. Temperature changes ≲1 °C mark efficient energy transfer. Low power oscillators, such as QCO, suffice to establish resonance in TLS systems. Although more delicate to set up than classical sonochemistry in the bulk,^24^ the advantages of substantial amplification without cavitation and low energy transducers in TLS are substantial.

## Appendix

### Statement of the Mathematical Problem

Let *k* (rad s^–1^) be the damping coefficient, *L* (m) the length of the thin layer, *c* (m s^–1^) the speed of sound in the fluid, and *h*(*t*) (Pa) the sound pressure from the transducer. The problem statement is

A.1

A.2

A.3

where *u*(*x*, *t*) is the sound pressure (Pa). It is useful to trade the forcing term at the boundary for an inhomogeneous equation. Consider

A.4

which satisfies the equation

The boundary and initial conditions are specified.

Note that *h*(0) and *h*′(0) are constant, and if either *h*(*t*) is a function of sin or cos, then either *h*(0) or *h*′(0) is zero, but not both. The system to solve is then,

A.5

A.6

A.7

where

## Solution of the PDE by Eigenfunction Expansion

Assume that the solution can be separated as *v*(*x*, *t*) and that *q*(*x*, *t*) can be similarly expanded.

A.8

A.9

On substitution of eq A.8 into eq A.5,

A.10

With −ϕ″(*x*) = λϕ(*x*) (see the SI) and , it follows that

Equations A.10 and A.9 are equated to yield the family of ODE’s.

A.11

The general solution is presented in the SI.

A.12

where

A.13

The coefficients *A*_*n*_ and *B*_*n*_ are such that *v*(*x*, *t*) satisfies *v*(*x*, 0) = *f*(*x*) and *v*_*t*_(*x*, 0) = *g*(*x*). By expansion of the initial conditions,

where

A.14

The notation

It can be shown that

A derivative of *v* is required to apply the second initial condition,

Let .

so that

A.15

A particular solution to the inhomogeneous equation

A.16

is obtained by Laplace transform. See the SI. The solution to eq A.5 is finally

A.17

where

A.18

The calculation of coefficients *A*_*n*_ and *B*_*n*_ and the calculation of *R*_*n*_(*t*) are shown in the SI. The original solution *u*(*x*, *t*), is then just

A.19

Further details are presented in the Supporting Information.

## Supporting Information Available

Details of mathematical solutions to complement the Appendix (PDF)

## Dedication

Parr and Leddy thank our late colleague Chet Duda for his excellent experimental work that demonstrated that thin layer sonochemistry is possible and allowed the thin layer sonochemistry model to be vetted.
