# Supplementary material for: Why Sonochemistry in a Thin Layer? Constructive Interference
Source: J Phys Chem C Nanomater Interfaces. 2023 Jun 16;127(25):12184–93. doi: 10.1021/acs.jpcc.3c00804 (PMC10320778; doi:10.1021/acs.jpcc.3c00804)
Supplement: Supplementary file 1 — jp3c00804_si_001.pdf [file jp3c00804_si_001.pdf]

Supplemental Information for

Why Sonochemistry in a Thin Layer? Constructive Interference.

Daniel L. Parr IV, Chester G. Duda, Johna Leddy*

University of Iowa
Department of Chemistry
Iowa City, IA 52240 USA
johna-leddy@uiowa.edu

Abstract

Sonochemistry undertaken in a thin layer of fluid has advantages of no visible cavitation, no turbulence, negligible temperature changes ($\lesssim 1\ ^{\circ}$C), use of low power transducers, and transmissibility (sound pressure amplification) of $\gtrsim 10^6$. Unlike sonochemistry in semi-infinite bulk fluids, resonance and so constructive interference of sound pressure can be established in a thin fluid layer. Constructive interference enables substantial amplification of sound pressure at the solid fluid interface. Fluid properties of sound velocity and attenuation, input frequency from the oscillator, and thickness of the thin fluid layer couples to established resonance for underdamped conditions. In thin layer sonochemistry (TLS), thin layers are established where the ultrasonic wavelength and oscillator-interface separation are comparable, on the order of a centimeter in water. A one dimensional wave equation model is solved to identify explicit relationships between system parameters that are required to establish resonance and constructive interference in a thin layer.

Supplemental Information

Derivation of the model is detailed here.

SI.1.1 Determination of $\phi_n(x)$

The function $\phi_n(x)$ is defined by the boundary conditions on Equation (A.5) and so

$$-\phi'' = \lambda\phi, \quad \phi'(0) = \phi(L) = 0$$

with general solution

$$\phi(x) = a\sin(x\sqrt{\lambda}) + b\cos(x\sqrt{\lambda}) \tag{SI.1}$$

Application of the first initial condition gives

$$\phi(x)'|_{x=0} = a\sqrt{\lambda}\cos(0) - b\sqrt{\lambda}\sin(0) = a\sqrt{\lambda} = 0$$

and implies that $a = 0$. Application of the second initial condition gives,

$$\phi(x)|_{x=L} = \cos(L\sqrt{\lambda})$$

and implies that

$$\begin{aligned} L\sqrt{\lambda_n} &= \frac{(2n+1)\pi}{2} \\ \sqrt{\lambda_n} &= \frac{(2n+1)\pi}{2L} \\ \lambda_n &= \left[\frac{(2n+1)\pi}{2L}\right]^2 \end{aligned}$$

so that

$$\phi_n(x) = \cos\left(\frac{(2n+1)\pi x}{2L}\right) \quad n = 0, 1, 2, \ldots \tag{SI.2}$$

SI.1.2 ODE Problem

The general solution to ODEs of the form

$$y''(t) + ky'(t) + \omega_n^2 y(t) = x(t), \quad y(0) = a, \quad y'(0) = b. \tag{SI.3}$$

is, by superposition, a sum of the general solution to the homogeneous equation $y_c(t)$ and a particular solution to the inhomogeneous equation y_p.

Homogeneous Equation First, consider the general solution to the homogeneous equation,

$$y''(t) + ky'(t) + \omega_n^2 y(t) = 0, \quad y(0) = a, \quad y'(0) = b \tag{SI.4}$$

The characteristic equation is

$$r^2 + kr + \omega_n^2 = 0 \tag{SI.5}$$

with roots

$$\lambda_\pm = -\frac{k}{2} \pm \frac{\sqrt{k^2 - 4\omega_n^2}}{2} \tag{SI.6}$$

If $k^2 \leq 4\omega_n^2$ then the eigenvalues are imaginary. Let $\beta_n = \sqrt{\omega_n^2 - \frac{k^2}{4}}$, then the real and imaginary parts of the root are separated as

$$\begin{aligned}
-\frac{k}{2} \pm \frac{\sqrt{k^2 - 4\omega_n^2}}{2} &= -\frac{k}{2} \pm \sqrt{\frac{k^2}{4} - \omega_n^2} \\
&= -\frac{k}{2} \pm \sqrt{(-1)\left(\omega_n^2 - \frac{k^2}{4}\right)} \\
&= -\frac{k}{2} \pm i\sqrt{\omega_n^2 - \frac{k^2}{4}} \\
&= -\frac{k}{2} \pm i\beta_n
\end{aligned}$$

A general (but imaginary) solution to Equation (SI.4) is then,

$$\begin{aligned}
y_c(t) &= c_1 e^{\lambda_+ t} + c_2 e^{\lambda_- t} \\
&= c_1 e^{\left(-\frac{k}{2} + i\beta_n\right)t} + c_2 e^{\left(-\frac{k}{2} - i\beta_n\right)t} \\
&= c_1 e^{-\frac{kt}{2}} e^{i\beta_n t} + c_2 e^{-\frac{kt}{2}} e^{-i\beta_n t} \\
&= c_1 e^{-\frac{kt}{2}} \left[\cos(\beta_n t) + i\sin(\beta_n t)\right] + c_2 e^{-\frac{kt}{2}} \left[\cos(\beta_n t) - i\sin(\beta_n t)\right]
\end{aligned}$$

Let

$$y_1 = c_1 e^{-\frac{kt}{2}} \left[\cos(\beta t) + i\sin(\beta_n t)\right] \tag{SI.7}$$

$$y_2 = c_2 e^{-\frac{kt}{2}} \left[\cos(\beta t) - i\sin(\beta_n t)\right] \tag{SI.8}$$

To obtain a purely real solution consider first

$$\begin{aligned}
c_1 y_1 + c_2 y_2 &= e^{-\frac{kt}{2}} \left[\cos(\beta_n t) + i\sin(\beta_n t)\right] + e^{-\frac{kt}{2}} \left[\cos(\beta_n t) - i\sin(\beta_n t)\right] \\
&= c_1 e^{-\frac{kt}{2}} \left[2\cos(\beta_n t)\right]
\end{aligned}$$

Let $c_1 = \frac{1}{2}$ so that

$$c_1 y_1 + c_2 y_2 = e^{-\frac{kt}{2}} \cos(\beta_n t)$$

Now, for a second solution, consider

$$\begin{aligned}
c_1 y_1 - c_2 y_2 &= e^{-\frac{kt}{2}} \left[\cos(\beta_n t) - i\sin(\beta_n t)\right] + e^{-\frac{kt}{2}} \left[\cos(\beta_n t) - i\sin(\beta_n t)\right] \\
&= c_2 e^{-\frac{kt}{2}} \left[2i\sin(\beta_n t)\right]
\end{aligned}$$

Let $c_2 = \frac{-i}{2}$ so that

$$c_1 y_1 - c_2 y_2 = e^{-\frac{kt}{2}} \sin(\beta_n t)$$

The general (and purely real) solution to Equation (SI.4) is,

$$y_c(t) = e^{-\frac{kt}{2}} \left[A\cos(\beta_n t) + B\sin(\beta_n t)\right]$$

Inhomogeneous Equation A particular solution to the inhomogeneous equation

$$y''(t) + ky'^2 y(t) = x(t) \tag{SI.9}$$

is obtained by Laplace transform. Assume that $y(0) = y'(0) = 0$ and let $\mathcal{L}[y(t)]$ denote the Laplace transform of $y(t)$. Then

$$\mathcal{L}\left[y''(t) + ky'^2 y(t)\right] = \mathcal{L}\left[x(t)\right]$$
$$s^2 Y(s) + ksY(s) + \omega^2 Y(s) = X(s)$$

where $\mathcal{L}[y(t)] = Y(s)$ and $\mathcal{L}[x(t)] = X(s)$. Rearranging, we get that

$$Y(s) = \frac{X(s)}{s^2 + ks + \omega^2}. \tag{SI.10}$$

which is inverted via convolution. Partial fractions decomposition gives,

$$\frac{1}{s^2 + ks + \omega^2} = \frac{1}{(s-\lambda_1)(s-\lambda_2)}$$
$$\frac{1}{s^2 + ks + \omega^2} = \frac{a}{s-\lambda_1} + \frac{b}{s-\lambda_2}$$
$$1 = a(s-\lambda_2) + b(s-\lambda_1)$$

where λ_1, λ_2 are roots of the polynomial $s^2 + ks + \omega^2$. Note that this polynomial is identical to the characteristic equation in Equation (SI.5). If $s = \lambda_1$, then,

$$1 = a(\lambda_1 - \lambda_2) \Rightarrow a = \frac{1}{\lambda_1 - \lambda_2}$$
$$1 = b(\lambda_2 - \lambda_1) \Rightarrow b = \frac{1}{\lambda_2 - \lambda_1}$$

and

$$\begin{aligned}\mathcal{L}^{-1}\left[\frac{1}{s^2 + ks + \omega^2}\right] &= \mathcal{L}^{-1}\left[\frac{1}{(\lambda_1-\lambda_2)(s-\lambda_1)}\right] + \mathcal{L}^{-1}\left[\frac{1}{(\lambda_2-\lambda_1)(s-\lambda_2)}\right] \\ &= \frac{1}{\lambda_1-\lambda_2}e^{\lambda_1 t} + \frac{1}{\lambda_2-\lambda_1}e^{\lambda_2 t} \\ &= \frac{1}{\lambda_1-\lambda_2}e^{\lambda_1 t} - \frac{1}{\lambda_1-\lambda_2}e^{\lambda_2 t} \\ &= \frac{1}{\lambda_1-\lambda_2}\left[e^{\lambda_1 t} - e^{\lambda_2 t}\right]\end{aligned}$$

From Equation (SI.5), we have that

$$\lambda_{1,2} = -\frac{k}{2} \pm i\beta$$

so that

$$\begin{aligned}\frac{1}{\lambda_1-\lambda_2}\left[e^{\lambda_1 t} - e^{\lambda_2 t}\right] &= \left(-\frac{k}{2} + i\beta + \frac{k}{2} + i\beta\right)\left[e^{\left(-\frac{k}{2}+i\beta\right)t} - e^{\left(-\frac{k}{2}-i\beta\right)t}\right] \\ &= \frac{1}{2i\beta}\left[e^{-\frac{kt}{2}}e^{i\beta t} - e^{-\frac{kt}{2}}e^{-i\beta t}\right] \\ &= \frac{1}{2i\beta}e^{-\frac{kt}{2}}\left[e^{i\beta t} - e^{-i\beta t}\right] \\ &= \frac{1}{\beta}e^{-\frac{kt}{2}}\frac{e^{i\beta t} - e^{-i\beta t}}{2i} \\ &= \frac{1}{\beta}e^{-\frac{kt}{2}}\sin(\beta t)\end{aligned}$$

The solution to Equation (SI.9) is

$$y_p = \frac{1}{\beta}\int_0^t e^{-\frac{k(t-s)}{2}}\sin(\beta(t-s))x(s)ds \tag{SI.11}$$

by convolution.

SI.1.3 Calculation of Coefficients A_n and B_n for $h(t) = \sin(\omega t)$

Note that

$$\int_0^L x^2 \cos\left(\frac{(2n+1)\pi x}{2L}\right) dx = (-1)^n 2L^3 \frac{(2\pi n + \pi)^2 - 8}{(2\pi n + \pi)^3} \tag{SI.12}$$

Let

$$r_n = (-1)^n 2L^3 \frac{(2\pi n + \pi)^2 - 8}{(2\pi n + \pi)^3} \tag{SI.13}$$

then the coefficient A_n is computed as

$$\begin{aligned} A_n &= \frac{\langle f, \phi_n \rangle}{\langle \phi_n, \phi_n \rangle} && \text{(SI.14)} \\ &= \frac{2}{L} \int_0^L f(x)\phi_n(x) dx \\ &= \frac{2}{L} \int_0^L -h(0)\frac{x^2}{L^2} \cos\left(\frac{(2n+1)\pi x}{2L}\right) dx \\ &= -\frac{2h(0)}{L^3} \int_0^L x^2 \cos\left(\frac{(2n+1)\pi x}{2L}\right) dx \\ A_n &= -\frac{2h(0)r_n}{L^3} && \text{(SI.15)} \end{aligned}$$

With $G(x) = g(x) + \dfrac{kf(x)}{2}$, it follows that

$$\begin{aligned} \beta_n B_n &= \frac{\langle G, \phi_n \rangle}{\langle \phi_n, \phi_n \rangle} && \text{(SI.16)} \\ &= \frac{2}{L} \int_0^L G(x)\phi_n(x) dx \\ &= \frac{2}{L} \int_0^L \left(g(x) + \frac{kf(x)}{2}\right) \phi_n(x) dx \\ &= \frac{2}{L} \int_0^L g(x)\phi_n(x) dx + \frac{2}{L} \int_0^L \frac{kf(x)}{2} \phi_n(x) dx \\ &= \frac{2}{L} \int_0^L g(x)\phi_n(x) dx + \frac{k}{L} \int_0^L f(x)\phi_n(x) dx \\ &= \frac{2}{L} \int_0^L -h'(0)\frac{x^2}{L^2} \cos\left(\frac{(2n+1)\pi x}{2L}\right) dx - k\frac{h(0)r_n}{L^3} \\ &= -\frac{2h'(0)}{L^3} \int_0^L x^2 \cos\left(\frac{(2n+1)\pi x}{2L}\right) dx - k\frac{h(0)r_n}{L^3} \\ &= -\frac{2h'(0)r_n}{L^3} - k\frac{h(0)r_n}{L^3} \\ &= -\frac{r_n}{L^3}\left[2h'(0) + kh(0)\right] && \text{(SI.17)} \end{aligned}$$

SI.1.4 Calculation of $R_n(t)$

Consider the integral

$$R_n(t) = \frac{1}{\beta_n} \int_0^t e^{-\frac{k(t-s)}{2}} \sin(\beta(t-s)) q_n(s) ds. \tag{SI.18}$$

Recall that

$$q(x,t) = \sum_{n=1}^{\infty} q_n(t)\phi_n(x).$$

Then,

$$
\begin{aligned}
q_n(t) &= \frac{2}{L}\int_0^L q(x,t)\phi_n(x) \\
&= \frac{2}{L}\int_0^L \left(-h''(t)\frac{x^2}{L^2} - kh'(t)\frac{x^2}{L^2} + h(t)\frac{2c^2}{L^2}\right)\phi_n(x) \\
&= -\frac{2}{L}\int_0^L h''(t)\frac{x^2}{L^2}\phi_n(x)dx - \frac{2}{L}\int_0^L kh'(t)\frac{x^2}{L^2}\phi_n(x)dx + \frac{2}{L}\int_0^L h(t)\frac{2c^2}{L^2}\phi_n(x)dx \\
&= -\frac{2h''(t)}{L^3}\int_0^L x^2\phi_n(x)dx - \frac{2kh'(t)}{L^3}\int_0^L x^2\phi_n(x)dx + \frac{4c^2h(t)}{L^3}\int_0^L \phi_n(x)dx \\
&= -\frac{2h''(t)r_n}{L^3} - \frac{2kh'(t)r_n}{L^3} + \frac{4c^2h(t)r_n'}{L^3} \\
&= -\frac{2}{L^3}\left(h''(t)r_n + kh'(t)r_n - 2c^2h(t)r_n'\right)
\end{aligned}
$$

where

$$
r_n' = \frac{(-1)^n 2L}{(2n+1)\pi} \tag{SI.19}
$$

Then finally,

$$
\begin{aligned}
R_n\left(t\right) &= \frac{1}{\beta_n}\int_0^t e^{-\frac{k(t-s)}{2}}\sin(\beta_n(t-s))q_n(s)ds \\
&= \frac{1}{\beta_n}\int_0^t e^{-\frac{k(t-s)}{2}}\sin(\beta_n(t-s))\left(-\frac{2h''(s)r_n}{L^3} - \frac{2kh'(s)r_n}{L^3} + \frac{4c^2h(s)r_n'}{L^3}\right)ds \\
&= \frac{2}{\beta_n L^3}\int_0^t e^{-\frac{k(t-s)}{2}}\sin(\beta_n(t-s))\left(-h''(s)r_n - kh'(s)r_n + 2c^2h(s)r_n'\right)ds
\end{aligned}
$$

which gives

$$
\begin{aligned}
R_n\left(t\right) = -\frac{2r_n}{\beta_n L^3}\int_0^t e^{-\frac{k(t-s)}{2}}\sin(\beta_n(t-s))h''(s)ds - \frac{2kr_n}{\beta_n L^3}\int_0^t e^{-\frac{k(t-s)}{2}}\sin(\beta_n(t-s))h'(s)ds \\
+ \frac{4c^2r_n'}{\beta_n L^3}\int_0^t e^{-\frac{k(t-s)}{2}}\sin(\beta_n(t-s))h(s)ds
\end{aligned} \tag{SI.20}
$$

This requires the calculation of three integrals that depend on $h(t)$ and so it is necessary to specify the input $h(t)$. Let

$$
h(t) = \sin(\omega t) \tag{SI.21}
$$

The necessary derivatives are

$$
h'(t) = \omega\cos(\omega t) \tag{SI.22}
$$

$$
h''^2\sin(\omega t) \tag{SI.23}
$$

$$
h'(0) = \omega \tag{SI.24}
$$

$$
h''(0) = 0 \tag{SI.25}
$$

It follows that

$$
R_n\left(t\right)(t) = \left(\frac{2r_n\omega^2}{\beta_n L^3} + \frac{4c^2r_n'}{\beta_n L^3}\right)\int_0^t e^{-\frac{k(t-s)}{2}}\sin(\beta_n(t-s))\sin(\omega s)ds \tag{SI.26}
$$

$$
-\frac{2kr_n\omega}{\beta_n L^3}\int_0^t e^{-\frac{k(t-s)}{2}}\sin(\beta_n(t-s))\cos(\omega s)ds \tag{SI.27}
$$

$$
= \left(\frac{2r_n\omega^2}{\beta_n L^3} + \frac{4c^2r_n'}{\beta_n L^3}\right)I_1(t) - \frac{2kr_n\omega}{\beta_n L^3}I_2(t) \tag{SI.28}
$$

SI.1.5 Calculation of I_1 and I_2

The calculation of integrals I_1 and I_2 is shown.

Calculation of I_1 Consider the first integral

$$I_1 = \int_0^t e^{\frac{-k(t-s)}{2}} \sin(\beta_m(t-s))\sin(\omega_m s)ds.$$

Let $u = t - s$. Then

$$I_1 = \int_0^t e^{\frac{-ku}{2}} \sin(\beta_m u)\sin(\omega_m(t-u))du \tag{SI.29}$$

where

$$\begin{aligned}\sin(\beta_m u)\sin(\omega_m(t-u)) &= \frac{e^{i\beta_m u} - e^{-i\beta_m u}}{2i} \times \frac{e^{i\omega_m(t-u)} - e^{-i\omega_m(t-u)}}{2i} \\ &= -\frac{1}{2}\cos(\beta_m u + \omega_m(t-u)) + \frac{1}{2}\cos(\beta_m u - \omega_m(t-u)) \\ &= \frac{1}{2}\cos(\beta_m u - \omega_m(t-u)) - \frac{1}{2}\cos(\beta_m u + \omega_m(t-u))\end{aligned}$$

Further let $\alpha_1(u) = \beta_m u - \omega_m(t-u)$ and $\alpha_2 = \beta_m u + \omega_m(t-u)$ where $\alpha_1' = \beta_m + \omega_m$ and $\alpha_2' = \beta_m - \omega_m$. Then,

$$\sin(\beta_m u)\sin(\omega_m(t-u)) = \frac{1}{2}\cos(\alpha_1) - \frac{1}{2}\cos(\alpha_2) \tag{SI.30}$$

and

$$I_{1,\alpha} = \frac{1}{2}\int_0^t e^{\frac{-ku}{2}} \cos(\alpha(u))du$$

A first application of integration by parts with

$$\begin{aligned} u &= \cos(\alpha(u)) \\ du &= -\alpha' \sin(\alpha(u))du \\ v &= -\frac{2}{k}e^{\frac{-ku}{2}} \\ dv &= e^{\frac{-ku}{2}}du \end{aligned}$$

gives

$$\begin{aligned} I_{1,\alpha} &= \frac{1}{2}\int_0^t e^{\frac{-ku}{2}} \cos(\alpha(u))du \\ &= \frac{1}{2}\left[-\frac{2}{k}e^{\frac{-ku}{2}} \cos(\alpha(u))\Bigg|_{u=0}^{u=t}\right] - \frac{\alpha'}{2}\int_0^t \frac{2}{k}e^{\frac{-ku}{2}} \sin(\alpha(u))du \\ &= -\frac{1}{k}e^{\frac{-ku}{2}} \cos(\alpha(u))\Bigg|_{u=0}^{u=t} - \frac{\alpha'}{k}\int_0^t e^{\frac{-ku}{2}} \sin(\alpha(u))du \end{aligned}$$

A second integration by parts with

$$\begin{aligned} u &= \sin(\alpha(u)) \\ du &= \alpha' \cos(\alpha(u)) \\ v &= -\frac{2}{k}e^{\frac{-ku}{2}} \\ dv &= e^{\frac{-ku}{2}}du \end{aligned}$$

gives $I_{1,\alpha}$.

$$\begin{aligned} I_{1,\alpha} &= -\frac{\alpha'}{k}\left[-\frac{2}{k}e^{\frac{-ku}{2}}\sin(\alpha(u))\Big|_{u=0}^{u=t} - \int_0^t -\frac{2}{k}e^{\frac{-ku}{2}}\alpha'\cos(\alpha(u))\right] \\ &= -\frac{\beta_m+\omega_m}{k}\left[-\frac{2}{k}e^{\frac{-ku}{2}}\sin(\beta_m u-\omega_m(t-u))\Big|_{u=0}^{u=t} + \frac{2\alpha'}{k}\int_0^t e^{\frac{-ku}{2}}\cos(\alpha(u))du\right] \\ &= \frac{2\alpha'}{k^2}e^{\frac{-ku}{2}}\sin(\alpha(u))\Big|_{u=0}^{u=t} - \frac{4(\alpha'^2}{k^2}I_{1a} \end{aligned}$$

Then,

$$\begin{aligned} I_{1,\alpha} &= -\frac{1}{k}e^{\frac{-ku}{2}}\cos(\alpha(u))\Big|_{u=0}^{u=t} + \frac{2\alpha'}{k^2}e^{\frac{-ku}{2}}\sin(\alpha(u))\Big|_{u=0}^{u=t} - \frac{4(\alpha'^2}{k^2}I_{1a} \\ I_{1,\alpha} + \frac{4(\alpha'^2}{k^2}I_{1,\alpha} &= -\frac{1}{k}e^{\frac{-ku}{2}}\cos(\alpha(u))\Big|_{u=0}^{u=t} + \frac{2\alpha'}{k^2}e^{\frac{-ku}{2}}\sin(\alpha(u))\Big|_{u=0}^{u=t} \\ I_{1,\alpha}\left(1+\frac{4(\alpha'^2}{k^2}\right) &= -\frac{1}{k}e^{\frac{-ku}{2}}\cos(\alpha(u))\Big|_{u=0}^{u=t} + \frac{2\alpha'}{k^2}e^{\frac{-ku}{2}}\sin(\alpha(u))\Big|_{u=0}^{u=t} \\ I_{1,\alpha} &= \frac{-\frac{1}{k}e^{\frac{-ku}{2}}\cos(\alpha(u))\Big|_{u=0}^{u=t} + \frac{2\alpha'}{k^2}e^{\frac{-ku}{2}}\sin(\alpha(u))\Big|_{u=0}^{u=t}}{\left(1+\frac{4(\alpha'^2}{k^2}\right)} \\ I_{1,\alpha} &= \frac{-\frac{1}{k}e^{\frac{-kt}{2}}\cos(\alpha(t)) + \frac{1}{k}\cos(\alpha(0)) + \frac{2\alpha'}{k^2}e^{\frac{-kt}{2}}\sin(\alpha(t)) - \frac{2\alpha'}{k^2}\sin(\alpha(0))}{\left(1+\frac{4(\alpha'^2}{k^2}\right)} \end{aligned}$$

Thus for any $\alpha(u)$,

$$I_{1,\alpha} = \frac{\frac{2\alpha'}{k^2}\left[e^{\frac{-kt}{2}}\sin(\alpha(t))-\sin(\alpha(0))\right] - \frac{1}{k}\left[e^{\frac{-kt}{2}}\cos(\alpha(t))-\cos(\alpha(0))\right]}{\left(1+4\left(\frac{\alpha'}{k}\right)^2\right)}$$

assuming that $k \neq 0$. Substitution of α_1 and α_2 for α gives the value of the two integrals,

$$I_{1,\alpha_1} = \frac{\frac{2(\beta_n+\omega)}{k^2}\left[e^{\frac{-kt}{2}}\sin(\beta_n t)+\sin(\omega t)\right] - \frac{1}{k}\left[e^{\frac{-kt}{2}}\cos(\beta_n t)-\cos(\omega t)\right]}{\left(1+4\left(\frac{\beta_n+\omega}{k}\right)^2\right)} \tag{SI.31}$$

$$I_{1,\alpha_2} = \frac{\frac{2(\beta_n-\omega)}{k^2}\left[e^{\frac{-kt}{2}}\sin(\beta_n t)-\sin(\omega t)\right] - \frac{1}{k}\left[e^{\frac{-kt}{2}}\cos(\beta_n t)-\cos(\omega t)\right]}{\left(1+4\left(\frac{\beta_n-\omega}{k}\right)^2\right)} \tag{SI.32}$$

so that

$$I_1(t) = I_{1,\alpha_1}(t) - I_{1,\alpha_2}(t) \tag{SI.33}$$

Calculation of I_2 Consider the second integral

$$I_2 = \int_0^t e^{\frac{-k(t-s)}{2}}\sin(\beta_m(t-s))\cos(\omega_m s)ds.$$

Again let $u = t-s$, then,

$$I_2 = \int_0^t e^{\frac{-ku}{2}}\sin(\beta_m u)\cos(\omega_m(t-u))du \tag{SI.34}$$

where

$$\sin(\beta_m u)\cos(\omega_m(t-u)) = \frac{e^{i\beta_m u} - e^{-i\beta_m u}}{2i} \times \frac{e^{i\omega_m(t-u)} + e^{-i\omega_m(t-u)}}{2}$$
$$= \frac{1}{2}\left[\sin(\beta_m u + \omega_m(t-u)) + \sin(\beta_m u - \omega_m(t-u))\right]$$
$$= \frac{1}{2}\sin(\beta_m u + \omega(t-u)) + \frac{1}{2}\sin(\beta_m u - \omega(t-u))$$
$$= \frac{1}{2}\sin(\alpha_1(u)) + \frac{1}{2}\sin(\alpha_2(u))$$

To calculate I_2, integrate by parts once with

$$u = \sin(\alpha(u))$$
$$du = \alpha' \cos(\alpha(u))du$$
$$v = -\frac{2}{k}e^{\frac{-ku}{2}}$$
$$dv = e^{\frac{-ku}{2}}du$$

to get

$$I_{2,\alpha} = \frac{1}{2}\int_0^t e^{\frac{-ku}{2}}\sin(\alpha(u))du$$
$$= \frac{1}{2}\left[-\frac{2}{k}e^{\frac{-ku}{2}}\sin(\alpha(u))\Big|_{u=0}^{u=t} - \int_0^t -\frac{2}{k}e^{\frac{-ku}{2}}\alpha'\cos(\alpha(u))du\right]$$
$$= -\frac{1}{k}e^{\frac{-ku}{2}}\sin(\alpha(u))\Big|_{u=0}^{u=t} + \frac{\alpha'}{k}\int_0^t e^{\frac{-ku}{2}}\cos(\alpha(u))du$$

A second integration by parts with

$$u = \cos(\alpha(u))$$
$$du = -\alpha'\sin(\alpha(u))$$
$$v = -\frac{2}{k}e^{\frac{-ku}{2}}$$
$$dv = e^{\frac{-ku}{2}}du$$

gives

$$\frac{\alpha'}{k}\int_0^t e^{\frac{-ku}{2}}\cos(\alpha(u))du = \frac{\alpha'}{k}\left[-\frac{2}{k}e^{\frac{-ku}{2}}\cos(\alpha(u))\Big|_{u=0}^{u=t} - \int_0^t -\frac{2}{k}e^{\frac{-ku}{2}}(-\alpha'\sin(\alpha(u)))du\right]$$
$$= \frac{\alpha'}{k}\left[-\frac{2}{k}e^{\frac{-ku}{2}}\cos(\alpha(u))\Big|_{u=0}^{u=t} - \frac{2\alpha'}{k}\int_0^t e^{\frac{-ku}{2}}\sin(\alpha(u))du\right]$$
$$= \left[-\frac{2\alpha'}{k^2}e^{\frac{-ku}{2}}\cos(\alpha(u))\Big|_{u=0}^{u=t} - 2\left(\frac{\alpha'}{k}\right)^2\int_0^t e^{\frac{-ku}{2}}\sin(\alpha(u))du\right]$$
$$= -\frac{2\alpha'}{k^2}e^{\frac{-ku}{2}}\cos(\alpha(u))\Big|_{u=0}^{u=t} - 4\left(\frac{\alpha'}{k}\right)^2 I_{2,\alpha}$$

Finally, rearrangement to solve for $I_{2,\alpha}$ gives

$$I_{2,\alpha} = -\frac{1}{k}e^{\frac{-ku}{2}}\sin(\alpha(u))\Big|_{u=0}^{u=t} + -\frac{2\alpha'}{k^2}e^{\frac{-ku}{2}}\cos(\alpha(u))\Big|_{u=0}^{u=t} - 4\left(\frac{\alpha'}{k}\right)^2 I_{2,\alpha}$$

$$I_{2,\alpha} + 4\left(\frac{\alpha'}{k}\right)^2 I_{2,\alpha} = -\frac{1}{k}e^{\frac{-ku}{2}}\sin(\alpha(u))\Big|_{u=0}^{u=t} + -\frac{2\alpha'}{k^2}e^{\frac{-ku}{2}}\cos(\alpha(u))\Big|_{u=0}^{u=t}$$

$$I_{2,\alpha}\left(1 + 4\left(\frac{\alpha'}{k}\right)^2\right) = -\frac{1}{k}e^{\frac{-ku}{2}}\sin(\alpha(u))\Big|_{u=0}^{u=t} + -\frac{2\alpha'}{k^2}e^{\frac{-ku}{2}}\cos(\alpha(u))\Big|_{u=0}^{u=t}$$

$$I_{2,\alpha} = \frac{-\frac{1}{k}e^{\frac{-ku}{2}}\sin(\alpha(u))\Big|_{u=0}^{u=t} + -\frac{2\alpha'}{k^2}e^{\frac{-ku}{2}}\cos(\alpha(u))\Big|_{u=0}^{u=t}}{\left(1 + 4\left(\frac{\alpha'}{k}\right)^2\right)}$$

$$I_{2,\alpha} = \frac{-\frac{1}{k}e^{\frac{-kt}{2}}\sin(\alpha(t)) + \frac{1}{k}\sin(\alpha(0)) + -\frac{2\alpha'}{k^2}e^{\frac{-kt}{2}}\cos(\alpha(t)) + \frac{2\alpha'}{k^2}\cos(\alpha(0))}{\left(1 + 4\left(\frac{\alpha'}{k}\right)^2\right)}$$

$$I_{2,\alpha} = \frac{-\frac{1}{k}\left[e^{\frac{-kt}{2}}\sin(\alpha(t)) - \sin(\alpha(0))\right] - \frac{2\alpha'}{k^2}\left[e^{\frac{-kt}{2}}\cos(\alpha(t)) - \cos(\alpha(0))\right]}{\left(1 + 4\left(\frac{\alpha'}{k}\right)^2\right)} \tag{SI.35}$$

$$I_{2,\alpha_1} = \frac{-\frac{1}{k}\left[e^{\frac{-kt}{2}}\sin(\beta_m t) + \sin(\omega_m t)\right] - \frac{2(\beta_m+\omega_m)}{k^2}\left[e^{\frac{-kt}{2}}\cos(\beta_m t) - \cos(\omega_m t)\right]}{\left(1 + 4\left(\frac{\beta_m+\omega_m}{k}\right)^2\right)} \tag{SI.36}$$

$$I_{2,\alpha_2} = \frac{-\frac{1}{k}\left[e^{\frac{-kt}{2}}\sin(\beta_m t) - \sin(\omega_m t)\right] - \frac{2(\beta_m-\omega_m)}{k^2}\left[e^{\frac{-kt}{2}}\cos(\beta_m t) - \cos(\omega_m t)\right]}{\left(1 + 4\left(\frac{\beta_m-\omega_m}{k}\right)^2\right)} \tag{SI.37}$$

so that finally

$$I_2(t) = I_{2,\alpha_1}(t) + I_{2,\alpha_2}(t) \tag{SI.38}$$
